# The role of death domain proteins in host response upon SARS-CoV-2 infection: modulation of programmed cell death and translational applications

**DOI:** 10.1038/s41420-020-00331-w

**Published:** 2020-10-10

**Authors:** Nikita V. Ivanisenko, Kamil Seyrek, Nikolay A. Kolchanov, Vladimir A. Ivanisenko, Inna N. Lavrik

**Affiliations:** 1 The Federal Research Center Institute of Cytology and Genetics SB RAS, Novosibirsk, Russia; 2 Novosibirsk State University, Novosibirsk, Russia; 3 Translational Inflammation Research, CDS, Medical Faculty, Otto von Guericke University, Magdeburg, Germany

**Keywords:** Cell death, Infection, Inflammasome

## Abstract

The current pandemic of novel severe acute respiratory syndrome coronavirus (SARS-CoV-2) poses a significant global public health threat. While urgent regulatory measures in control of the rapid spread of this virus are essential, scientists around the world have quickly engaged in this battle by studying the molecular mechanisms and searching for effective therapeutic strategies against this deadly disease. At present, the exact mechanisms of programmed cell death upon SARS-CoV-2 infection remain to be elucidated, though there is increasing evidence suggesting that cell death pathways play a key role in SARS-CoV-2 infection. There are several types of programmed cell death, including apoptosis, pyroptosis, and necroptosis. These distinct programs are largely controlled by the proteins of the death domain (DD) superfamily, which play an important role in viral pathogenesis and host antiviral response. Many viruses have acquired the capability to subvert the program of cell death and evade the host immune response, mainly by virally encoded gene products that control cell signaling networks. In this mini-review, we will focus on SARS-CoV-2, and discuss the implication of restraining the DD-mediated signaling network to potentially suppress viral replication and reduce tissue damage.

## Facts

- There is an increasing evidence that cell death pathways play a key role in SARS-CoV-2 infection.
- Cell death and inflammation pathways are largely controlled by the proteins of the DD superfamily.
- The DD superfamily comprises the DD, death effector domain (DED), caspase activation recruitment domain (CARD), and pyrin domain (PYD) subfamilies.

## Open questions

- Restraining of SARS-CoV-2 viral replication and virus-induced tissue damage through modulation of DD network might pave the way toward new therapeutic approaches.
- Understanding the different stoichiometry of DD, DED, PYD, and CARD macromolecular platforms might provide the link toward uncovering the cross talk between these pathways.
- The role of positive and negative regulation of programmed cell death pathways by SARS-CoV-2 infection in the viral spread.
- Targeting the DD superfamily proteins might play a pivotal role in the inflammation control during SARS-CoV-2 infection.

## Introduction

During the end of 2019 and the beginning of 2020, multiple human cases of novel coronavirus infection were reported, caused by severe acute respiratory syndrome coronavirus (SARS-CoV-2). Although some initial cases were linked to a local seafood market in Wuhan, its origin, intermediate hosts, and how it was transmitted to humans are still largely unknown. Coronaviruses (CoVs) are enveloped, nonsegmented, positive-sense single-stranded RNA viruses^1,2^. With genomes ranging in size from 26 to 32 kilobases, they are among the largest known viral RNA (vRNA) genomes. The virion has a nucleocapsid composed of genomic RNA and phosphorylated nucleocapsid (N) protein, which is buried inside phospholipid bilayers and covered by two different types of spike proteins: the spike glycoprotein trimmer (S) that can be found in all CoVs, and the hemagglutinin–esterase that exists in some CoVs. The membrane (M) protein (a type III transmembrane glycoprotein) and the envelope (E) protein are located among the S proteins in the virus envelope. SARS-CoV-2 as well as earlier reported SARS-CoV and Middle East respiratory syndrome coronavirus (MERS-CoV) infections may remain asymptomatic in the early stage, until causing severe pneumonia, dyspnea, renal insufficiency, and even death^3,4^. Currently, no promising antiviral treatment is available. There are, however, numerous compounds with proven effectiveness against SARS-CoV and MERS-CoV, which have not yet been tested for the newly emerged SARS-CoV-2.

There are several types of regulated cell death^5^. Apoptosis is a form of regulated cell death that eliminates damaged and excessive cells to maintain tissue homeostasis. There are two key ways of apoptosis induction: the intrinsic and the extrinsic pathway^6^. The intrinsic pathway is initiated via mitochondrial outer membrane permeabilisation, which is followed by the release of several proapoptotic factors from the mitochondria, resulting in the initiation of the cell death cascade^5^. The extrinsic cell death pathway is triggered by the stimulation of the death receptors (DRs)^7,8^. Six DR family members have been characterized so far: TNF-R1, CD95 (FAS/APO-1), DR3, TRAIL-R1/DR4, TRAIL-R2/DR5, and DR6^9^. All members of the DR family are characterized by the presence of the death domain (DD) playing the key role in the transduction of apoptotic and antiapoptotic signals^7,10,11^.

**Fig. 1:**
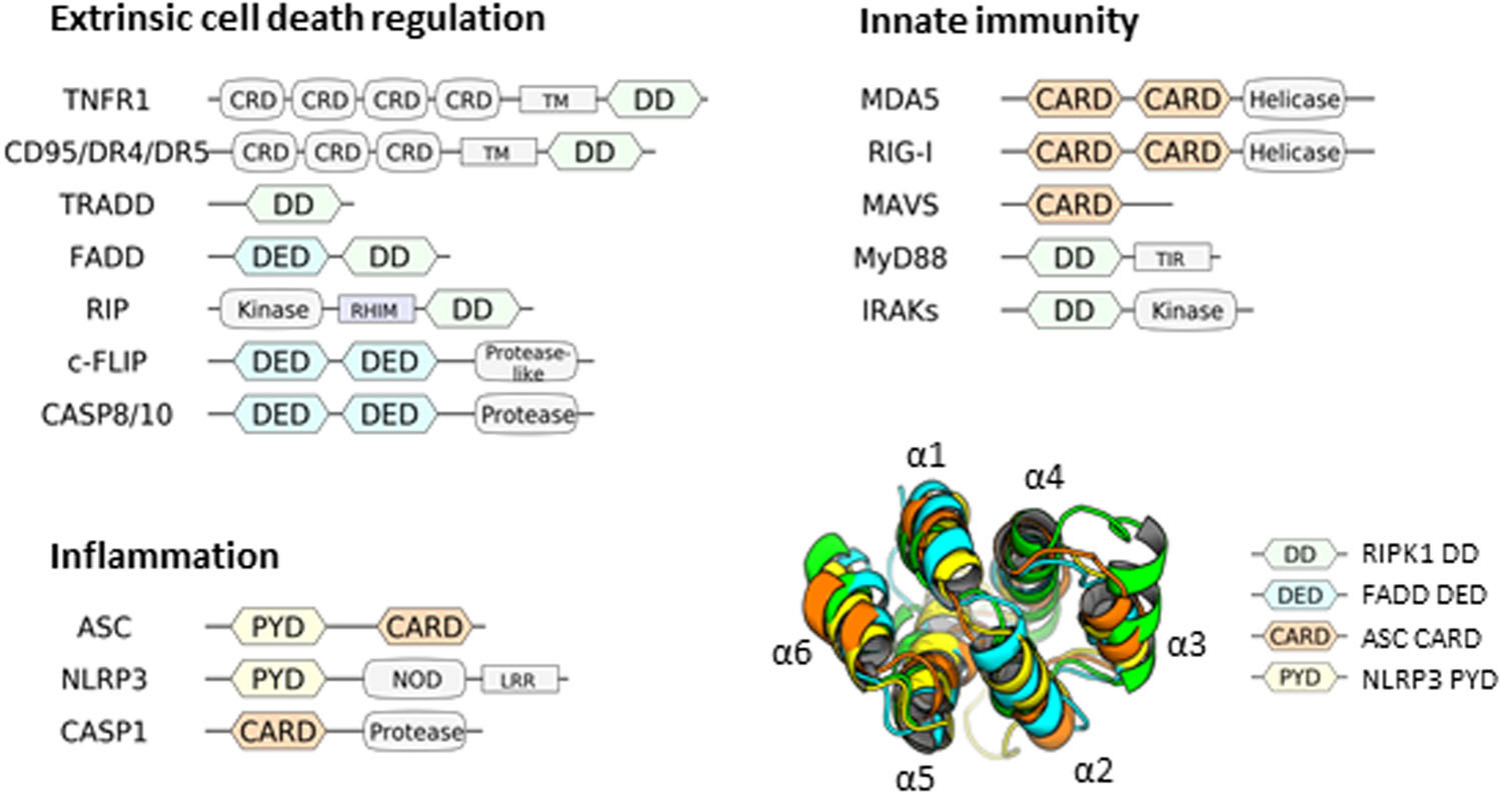
Domain organization of the major DD superfamily members controlling inflammation, cell death, and the innate immune response. DD, CARD, DED, and PYD are shown in green, blue, yellow, and orange, respectively. The superimposed representative structures of DDs of RIPK1 DD (PDB identifier 6AC5), FADD DED (PDB identifier 1A1W), ASC CARD (PDB identifier 5GPQ), and NLRP3 PYD (PDB identifier 3QF2) are shown. N- and C-terminal regions were truncated to improve visual perception.

The DD family is a subfamily of the so-called DD superfamily^9,12^. The DD superfamily shares a highly conservative structural fold that includes six to seven α-helices (Fig. 1). The DD superfamily comprises the DD, death effector domain (DED), caspase activation recruitment domain (CARD), and pyrin domain (PYD) subfamilies (Fig. 1). The members of this superfamily are capable of assembling into oligomeric structures that are formed via homotypic interactions between DD superfamily members^13^. In this way, the regulation of programmed cell death, inflammation, and host defense against intracellular pathogens is largely controlled by the proteins from the DD superfamily^12^. In particular, the key regulatory step involves the assembly of macromolecular complexes, including death-inducing signaling complex (DISC), necrosome, ripoptosome, faddosome, myddosome, and PIDDosome that serve as platforms for the initiation of a particular signaling pathway^14–22^. The individual function and sequence of proteins from the DD superfamily likely diverged in the course of evolution to provide multiple strategies for host defense and programmed cell death activation. However, at the same time, the cross talk between these pathways through the proteins of DD superfamily has gained more attention recently. It turned out that the key players of the extrinsic apoptosis pathway, such as Fas-associated protein with DD (FADD), cellular FLICE inhibitory protein (c-FLIP), caspase-8, and RIPK1 play the major role in the regulation of different programs of cell death, including apoptosis, necroptosis, and pyroptosis, as well as innate immune responses^12,17,23–26^. Thus, targeting the DD superfamily proteins of the extrinsic pathway might play a pivotal role in the inflammation control during the viral infection and restore the innate immune response.

Host cells eliminate virally infected cells via cell death pathways, including apoptosis and necroptosis, which aborts virus infection^27^. On the other hand, some viruses take advantage of inducing cell death as a way to release and disseminate progeny viruses or as a strategy to evade the immune system^28^. Activation of the necroptosis response in host cells, in turn can lead to antiviral inflammation^29^. In this review, we will mainly focus on the restraining of SARS-CoV-2 viral replication and virus-induced tissue damage through modulation of the signaling pathways mediated by DD superfamily members, and possible treatment therapies.

## Targeting extrinsic cell death as a strategy to combat SARS-CoV-2 replication at an early stage of the infection

**Fig. 2:**
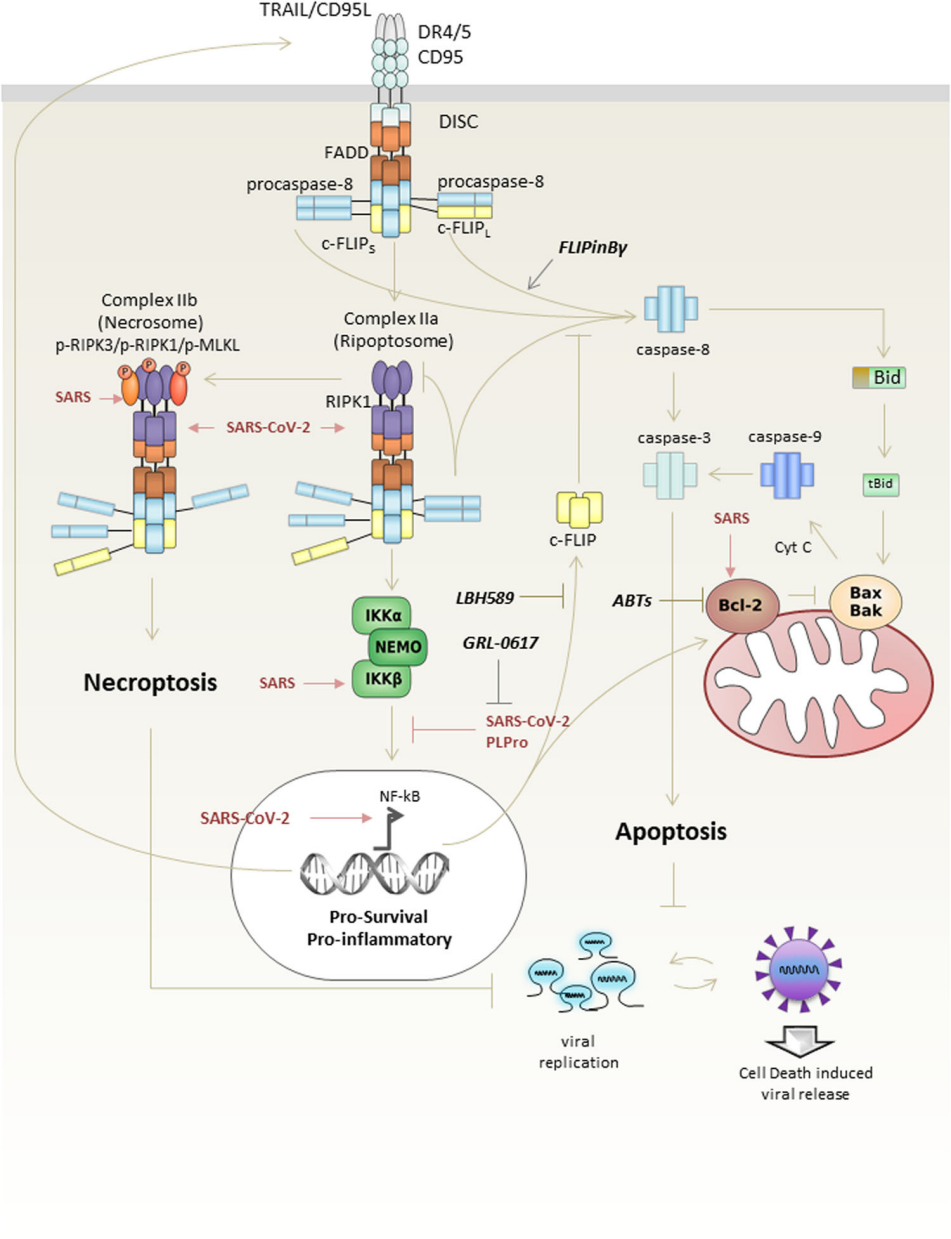
Induction of cell death via DR activation and SARS/SARS-CoV-2 infection. The key signaling platforms involved in programmed cell death through CD95 and DR4/5, as well as their cross talk are shown. The proteins that are regulated by SARS and SARS-CoV-2 infections are denoted in purple. Small-molecule targets are indicated with black arrows.

The extrinsic cell death pathway is triggered via DRs^9^. Activation of CD95 or TRAIL-R1/2 with cognate ligands or agonistic antibodies results in the formation of the DISC at the cellular membrane^20^. DISC consists of oligomerized receptors, the adaptor protein FADD, initiator zymogens procaspase-8 and -10, and the c-FLIP (Fig. 2). All interactions at the DISC are based on the intricate network of DD and DED interactions. DDs provide the basis for FADD and DR interactions, while DEDs are essential for the formation of the so-called DED filaments that de facto present the platform for procaspase-8 dimerization and activation^30–32^. Procaspase-8 activation at the DED filaments is inhibited by c-FLIP proteins^33^. One long and two short c-FLIP isoforms, named Long (L), Short (S), and Raji (R), i.e., c-FLIP_L_, c-FLIP_S_, and c-FLIP_R_, were reported^34^. c-FLIP_L_ at the DISC can act both in a proapoptotic as well as in an antiapoptotic manner^35,36^. The proapoptotic function of c-FLIP_L_ is mediated by the formation of catalytically active procaspase-8/c-FLIP_L_ heterodimers^37,38^. Short c-FLIP isoforms, c-FLIP_S_ and c-FLIP_R,_ act solely in an antiapoptotic manner^34^. Upon CD95 or TRAIL-R1/2 stimulation, procaspase-8 might be activated in other complexes, in particular, in the complex IIa or ripoptosome^17,18^ (Fig. 2). This complex is formed upon deubiquitinylation of another key protein of the DD networks—RIPK1, and similar to DISC comprises DED proteins: FADD, procaspase-8/10, and c-FLIP. Upon stimulation of the DRs and inhibition of caspase-8, RIPK1 serves as a core for the formation of the complex IIb or necrosome promoting necroptosis^39,40^ (Fig. 2). This macromolecular platform comprises RIPK1, RIPK3, FADD, procaspase-8/10, and c-FLIP. Strikingly, stimulation of CD95 or TRAIL-R1/2 can also lead to the induction of non cell death pathways, including the induction of transcription factor NF-kB^24,41,42^.

*Coronaviridae* cause various diseases, including bronchitis, gastroenteritis, hepatitis, and systemic diseases on humans, bats, rodents, animals, and birds. Both host and viral factors affect coronavirus virulence and the disease severity in animals, which has allowed to study the key mechanisms of virus influence on cell death networks. For instance, it has been shown that infection of cell lines with canine coronavirus type II (CCoV-II) controls both intrinsic and extrinsic apoptosis to promote the replication of the virus^43,44^. Strikingly, one of the key mechanisms of virus replication control involves the inhibition of extrinsic apoptosis at early stages of infection via c-FLIP proteins^44^. c-FLIP expression is suppressed by the forkhead transcription factor FOXO3A^45,46^. The level of FOXO3A mRNA was found to be increased 12 h after CCoV-II infection, which might lead to triggering extrinsic apoptosis^44^. Moreover, CCoV-II infection is characterized by elevated levels of TRAILR 12 h post infection, suggesting an important role for the extrinsic apoptosis pathway at the later stages of canine coronavirus infection^44^.

Similar mechanisms might be involved in SARS-CoV-2 infections. Inducing extrinsic apoptosis at the early stage of infection might impair virus replication, and therefore this response might be counteracted by the virus through c-FLIP upregulation. SARS-CoV-2 virus can take advantage of this c-FLIP-mediated cell death delay for its own replication. Indeed, the most recent studies have demonstrated transcriptional changes for the key regulatory proteins and miRNAs of the DR pathways upon SARS-CoV-2 infection^47–49^. In particular, the upregulation of miRNA-155 was reported, which along with other targets represses FOXO3A that is a key suppressor of c-FLIP expression^50^. Remarkably, the c-FLIP expression level was found to be enhanced in several SARS-CoV-2-infected cell lines, together with key mediators of the DR signaling networks, such as A20 and DR5^47^. Elevated c-FLIP expression was observed at very early time points after SARS-CoV-2 infection, as well as in postmortal lung biopsies of COVID-19 patients. In line with these data, it is logical to assume that blocking extrinsic apoptosis via upregulation of c-FLIP proteins is one of the ways for SARS-CoV-2 to sustain its replication.

Besides c-FLIP protein, other targets of SARS-CoV-2 in the DR network are currently being uncovered. The upregulation of DR5 and A20 mRNAs, which is mentioned above, probably plays a key role at the later stages of infection by triggering cell death (Fig. 2). Likewise, using proteomic analysis, it was shown that SARS-CoV-2 directly interacts with RIPK1, one of the major components of DR complexes, e.g., complex IIa/ripoptosome or IIb/necrosome^2^. However, the role of this interaction in the viral life cycle remains to be elucidated. It might control apoptosis or necroptosis induction at the complex IIa or IIb, respectively, at the later stages of the viral response. Accordingly, SARS-CoV infection was able to target RIPK3, another key component of complex IIb, facilitating necroptotic cell, which might be similar for SARS-CoV-2^51^.

The delayed apoptosis of host cells is of advantage for the replication of coronavirus and aggravating the infection at its early stages. Therefore, inducing extrinsic apoptosis and caspase-8 activation via targeting c-FLIP or caspase-8 at the beginning of the infection seems to be an important strategy to inhibit virus replication, and limit the number of viruses. In this regard, recent reports on small molecules activating caspase-8 via targeting caspase-8/c-FLIP_L_ heterodimers, or small molecules activating caspase-8 at the initial steps after death ligand administration might be of particular interest^52^ (Fig. 2). In light of this hypothesis, the application of histone deacetylase inhibitors, such as LBH589, that have been accepted as effective agents against c-FLIP, presents an additional promising strategy^53^ (Fig. 2). Broad-spectrum antivirals, for instance, dsRNA-activated caspase oligomerizer, which selectively induces apoptosis in virus-containing host cells, also can be evaluated for their effectiveness against SARS-CoV-2^54^.

As mentioned above, coronaviruses also control the intrinsic apoptosis pathway. SARS Orf7a was shown to interact with antiapoptotic members of the Bcl-2 family, which might facilitate apoptosis at the later infection stages^55^. Bcl-xL was reported to play a role in suppression of proapoptotic activities of SARS in T cells^56^. The putative role of similar regulation in SARS-CoV-2 infection is yet to be studied. Moreover, the small-molecule ABT-263 targeting Bcl-2/Bcl-XL was reported to have antiviral properties and facilitate apoptosis induction upon infection, with several DNA and RNA viruses, including MERS-CoV^57^. It makes targeting Bcl-2 proteins another promising strategy to sensitize cells infected with SARS-CoV-2 to apoptosis.

The NF-κB pathway is considered as a major antiapoptotic pathway^24,47^. Activation of NF-κB leads to the upregulation of the most important apoptosis inhibitors, such as c-FLIP, antiapoptotic Bcl-2 family members, and XIAPs. According to the analysis of mRNA expression, several key components of the NF-κB pathway were shown to be upregulated upon SARS-CoV-2 infection^47–49^ (Fig. 2). Along with other NF-κB targets, increased expression of inhibitors of the apoptotic pathway, such as c-FLIP and XIAPs, was detected in this analysis. Moreover, according to recent proteomic analyses, multiple protein products of genes from the NF-κB pathway were shown to interact with SARS-CoV-2 proteins^2^. Hence, selective targeting of the NF-κB machinery in combination with inducing apoptosis might pave the way toward development of specific strategies for down modulating the replication of SARS-CoV-2. In this regard, it has to be mentioned that both SARS-CoV and MERS-CoV were reported to have multiple mechanisms to regulate the NF-κB induction by facilitating its activation or, on the opposite, suppressing it^58^. These different ways of influencing the NF-κB pathway might play a role at different stages of the infection.

Hyperactivation of the NF-κB pathway is one classical feature of viral infections, and might be important for the early stages of SARS-CoV-2 infection to block apoptosis. The induction of NF-κB activity is reported to be mediated by SARS-CoV 3a and 7a, and nsp1 proteins^58^. Several small-molecule inhibitors developed to inhibit the NF-κB activation, such as proteasome inhibitors, represent another valuable strategy to target the antiapoptotic proinflammatory response induced by SARS-CoV-2 and induce apoptosis^59^.

The suppression of NF-κB activity seems to be a crucial step for viral infection in promoting cell death. Among several mechanisms of NF-κB suppression by SARS-CoV is the direct interaction of the SARS-CoV M protein with IKKβ, leading to suppression of the NF-κB activation^60^. The proteolytic activity of SARS-CoV papain-like protease (PLpro) protease was also found to repress the NF-κB activation by removing the K48-linked ubiquitin chains from IκBα^61,62^. The latter is a crucial step in the activation of NF-κB leading to the degradation of IκBα and nuclear translocation of NF-κB. Interestingly, SARS-CoV-2 PLpro was reported to be less active in deubiquitinating critical components of the NF-κB pathway compared to SARS PLpro, but more active in suppressing type I interferon responses^63^. Small-molecule inhibitors of PLpro were reported to be active against both SARS and SARS-CoV-2, including the compound GRL-0617^61,63^. Their potential for clinical use is currently being evaluated. In addition, it has to be mentioned that SARS-CoV-2 infection may regulate extrinsic cell death and NF-κB pathways through interferon regulatory factors (IRFs), and therefore small-molecule inhibitors of PLpro are of high interest as an additional point of interference with extrinsic cell death and NF-κB pathways.

Taken together, dual regulation of cell death and NF-κB activity can play important role in regulation of the proinflammatory response, controlling the programmed cell death at different time intervals of viral replication. In this regard, it is also striking that MERS-CoV has been reported to induce apoptosis in primary T lymphocytes^3^. Moreover, there is the first evidence of involvement of the CD95 pathway in SARS-CoV-2-infected lymphoid cells, which would suppress the immune system of SARS-CoV-2-infected patients. In this case, the opposite strategy of restraining apoptosis would be particularly helpful in the battle between the host immune system and SARS-CoV-2.

## Targeting DD-mediated networks at the later stages of SARS-CoV-2 infection

**Fig. 3:**
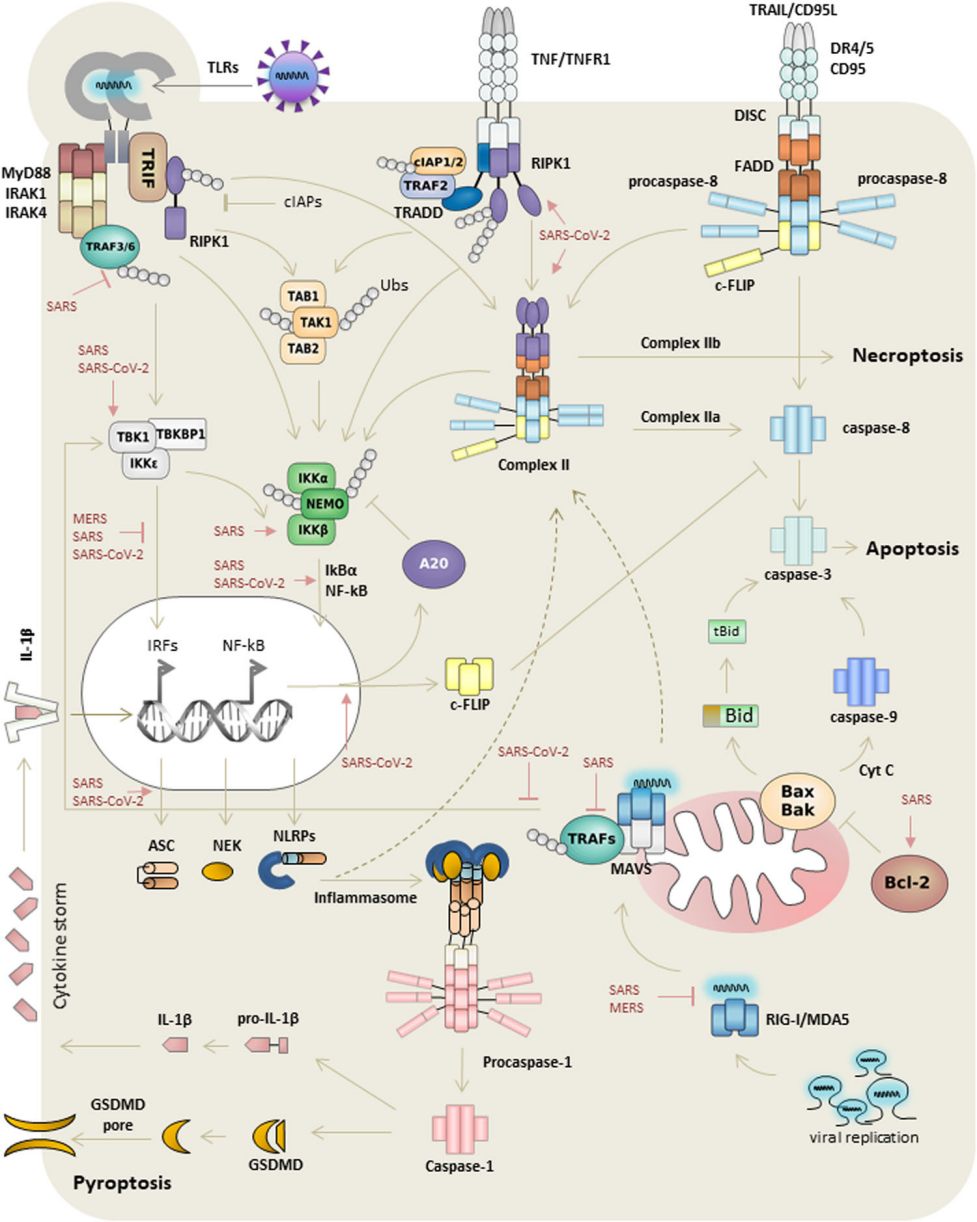
Influence of SARS, MERS, and SARS-Cov-2 infection on the DD network. The key signaling platforms comprising DD superfamily members are depicted, as well as their cross talk. The proteins that are regulated by SARS, MERS, and SARS-Cov-2 infection are denoted in purple.

Cytokine storm that occurs at the later stages of SARS-CoV-2 infection is reported to be one of the major reasons of high lethality due to the SARS-CoV-2 virus^64^. It is characterized by the release of the proinflammatory cytokines, such as TNF-α, IL6, and others. Another form of programmed cell death, termed pyroptosis, significantly contributes to the maturation of proinflammatory cytokines, such as IL-1β^65^. The maturation of IL-1β occurs upon assembly of the inflammasome, which serves as an initiator macromolecular platform in the pyroptosis pathway^66^ (Fig. 3). The inflammasome comprises pattern recognition receptors, including PYD-containing 3 (NLRP3), adaptor protein apoptosis-associated speck-like protein containing a CARD (ASC), and inflammatory procaspases, such as procaspase-1^65^. The assembly of the inflammasome is based on interactions between CARDs of procaspase-1 and ASC adaptor protein^12^. Inflammasome assembly leads to the autocatalytic activation of procaspase-1, and subsequent processing of pro-IL-1β and pro-IL-18 into their mature forms. A hyperproduction of proinflammatory cytokines leads to the cytokine storm that has been well-documented for SARS, and is considered to play a major role in SARS-CoV-2 infection as well^64^ (Fig. 3). In particular, for SARS-CoV infection, ORF and E proteins were reported to strongly facilitate NLRP3 inflammasome activation and similar mechanisms might play a role in SARS-CoV-2 infection^67,68^.

Furthermore, it might be suggested that reprogramming of pyroptosis through targeting the proteins of the extrinsic cell death pathway to induce different forms of programmed cell death may lead to a less pathogenic phenotype of SARS-CoV-2. This strategy can be achieved due to the tight cross talk between pyroptosis and apoptosis mediated by the members of extrinsic cell death pathways^25,26,69^. In particular, in recent studies it was shown that inflammasome adaptor ASC recruits procaspase-8 through a PYD/DED interaction^32^. Moreover, it was shown that catalytically inactive caspase-8 has a scaffold function in pyroptosis induction by promoting formation of ASC specks^25,26^. However, there are also studies indicating that the recruitment of procaspase-8 to the inflammasome results in apoptosis induction^70,71^. This proapoptotic activity is dependent on caspase-8 and negatively regulated by elevated c-FLIP levels. This further underlines the importance of compounds reducing c-FLIP levels, as promising agents against SARS-CoV-2 along with the compounds activating caspase-8^52,53^. Interestingly, c-FLIP has been recently shown to protect macrophages from LPS-induced pyroptosis via inhibition of complex II formation; thus, playing a dual role in pyroptosis and apoptosis regulation^72^. Accordingly, the strategy of increasing caspase-8 activity should certainly take into account the overall c-FLIP levels in the cell and their influence on caspase-8 activation.

vRNA is sensed by the innate immune system and triggers an antiviral response. Several members of Toll-like receptors (TLR) are involved in the recognition of viral dsRNA and innate immune system activation^73,74^. The major adaptor protein of TLR signaling is MyD88 that comprises DD. Thereby, MyD88 recruits the DD proteins IRAK4 and IRAK1 to form the oligomeric macromolecular complex termed Myddosome^75^. Myddosome allows proximity-induced phosphorylation of IRAKs, and their activation to trigger innate immunity pathway activation via TNF-receptor-associated factor 3 (TRAF3) and TRAF6^76^. The Myddosome pathway triggers the activation of a number of genes controlling the immune response (Fig. 3). Accordingly, it is not surprising that SARS-CoV-2 apparently developed multiple strategies to evade the Myddosome pathway (Fig. 3). In particular, SARS-CoV-2 was reported to target TBK1 and TBKBP1, key proteins involved in the activation of interferon-induced gene expression^2^. Importantly, similar mechanisms have been reported for MERS and SARS infections (Fig. 3). Moreover, SARS-CoV PLPro was reported to catalyze deubiquitination of TRAF3 and TRAF6, inhibiting the TLR7 innate immunity pathway^77^. This pathway is also the one leading to the suppression of IRF3 activation by MERS-CoV and SARS-CoV^27^. As mentioned above, small-molecule inhibitors of PLPro demonstrated potent activity against SARS-CoV and SARS-CoV-2 infection^61,63^, likely through complex activity including suppression of viral replication and restoration of the IRF3 pathway^63^.

Alternative to the Myddosome pathway, TLR3 and TLR4 receptors can engage TRADD via interactions with the TRIF domain^78^. This, in turn, results in the recruitment of RIPK1, likely through the formation of a DD platform that is capable of FADD and subsequent caspase-8/c-FLIP recruitment. This complex is reported to regulate inflammatory and cell death responses, and can be negatively modulated by the cellular inhibitor of apoptosis protein cIAP^79^. Interestingly, major proteins of RIPK1-dependent cell death activation were shown to be targeted by CoV proteins; however, there were no reports on the direct regulation of caspase-8-dependent cell death activation. This suggests that the modulation of caspase-8 activity in the complex formed through TLR3/TLR4 activation might also present a promising strategy for antiviral effects leading to apoptosis activation. Likewise, c-FLIP downregulation with the chemotherapeutic agent paclitaxel is reported to enhance the apoptotic cell death through TLR3 activation, which might imply a promising strategy^80^. Taken together, this further underlines the importance of developing compounds specifically increasing caspase-8 activity, also for the latter stages of the SARS-CoV-2 infection.

The DD, DED, and CARD interaction network also plays an important role in the intracellular dsRNA recognition pathway, which is mediated by RIG-I and MDA-5. Both RIG-I and MDA-5 comprise N-terminal CARDs that recruit MAVS through its CARD leading to the formation of macromolecular complex at the mitochondria. This complex also comprises DD- and DED-containing TRADD/RIPK1/caspase-8/FADD proteins leading to a macromolecular platform, which mediates the activation of IRF3 and NF-kB^81^. The MAVS/caspase-8 platform was shown to link RNA virus innate antiviral signaling to apoptosis^82^. The involvement of this pathway has been shown for SARS-CoV and MERS-CoV^27^, which allows to suggest an important role in SARS-CoV-2 infection as well. In line with this hypothesis, recent proteomics studies have demonstrated that MAVS might be indirectly regulated by SARS-CoV-2 through interactions with TOMM70^2^. The latter is a critical adaptor linking MAVS to the TBK/IRF3 complex at the mitochondria. Moreover, the role of DED proteins in the regulation of the innate immunity pathway suggests that targeting caspase-8 and c-FLIP represents another potential antiviral strategy. In this regard, an important role belongs to uncovering the distinct roles of procaspase-8 and c-FLIP in the activation of the intracellular dsRNA recognition pathway.

The development of therapeutic approaches based on the targeting the DD superfamily network is very important for combatting SARS-CoV-2 infection. At the early stages of infection, as discussed above, inducing apoptosis of infected cells via increasing caspase-8 activity, using small molecules or triggering apoptosis via TRAIL receptor agonists might be beneficial. In this regard, it might be hypothesized that a combinations of drugs can be derived from the clinical trials of TRAIL receptor agonists in cancer cells (Supplementary Table 1). Targeting the DD superfamily network plays a pivotal role in inflammation control during the later stages of SARS-CoV-2 infection. In this regard, a key role should belong to anti-inflammatory modulators, such as the above mentioned small-molecule inhibitors of PLpro and TNFα antagonists. The latter are in clinical trials performed by several groups (Table 1). In addition, the above discussed mechanisms of cross talk between apoptosis, pyroptosis, and inflammation via DD proteins have to be specially considered in future studies for the development of therapeutic approaches against SARS-CoV-2 infection.

**Table 1:** Clinical trials of TNFα inhibitors in COVID-19.

TNFα inhibitors	Number of patients	Combination	Clinical phase	Reference
Infliximab (Remicade, Kineret)	17	Premedication with Tylenol or diphenhydramine and prednisone	II	NCT04425538
Emapalumab (Gamifant)	54	None	II/III	NCT04324021
XPRO1595	366	None	II/III	NCT04370236

Taken together, DD superfamily members play a pivotal role in the formation of macromolecular platforms that initiate multiple signaling pathways from cell death to the inflammatory response. Moreover, the different stoichiometry of DD, DED, PYD, and CARD-containing proteins that provide the major core of these macromolecular platforms largely defines the cross talk between these pathways. Hence, targeting the key DD superfamily members presents a very important strategy in the development of treatment therapies for SARS-CoV-2 infections.

## Supplementary information

supplementary table
