# Supplementary material for: The role of death domain proteins in host response upon SARS-CoV-2 infection: modulation of programmed cell death and translational applications
Source: Cell Death Discov. 2020 Oct 10;6:101. doi: 10.1038/s41420-020-00331-w (PMC7547561; doi:10.1038/s41420-020-00331-w)
Supplement: Supplementary file 1 — supplementary table [file 41420_2020_331_MOESM1_ESM.docx]

**Supplementary Table 1: Clinical trials of TRAIL receptor agonists in cancer cells**

|  |  |  |  |  |
| --- | --- | --- | --- | --- |
| Recombinant form of TRAIL | **Combination** | **Cancer** | **Clinical phase** | **Reference** |
| Dulanermin / rhApo2L / AMG951 | 5-fluorouracil or CPT-11 | Colon, lung, breast, kidney, brain, and skin | Pre-clinical | ^1^ |
|  | Ritiuximab | Non-Hodgkin's lymphoma | Ib | ^2^ |
|  | Ritiuximab | Non-Hodgkin's lymphoma | Ib/II | ^3^ |
|  | Paclitaxel, carboplatin, and  bevacizumab | Advanced non-small cell lung cancer | Ib | ^4^ |
|  | Paclitaxel, carboplatin, and  bevacizumab | Advanced non-small cell lung cancer | II | ^5^ |
|  | Modified FOLFOX6 and  bevacizumab | Colorectal | Ib | ^6^ |
|  | None | Advanced solid tumours | Ia | ^7^ |
|  | None | Advanced tumours | Ia | ^8^ |
|  | Conatumumab (AMG  655) | Lung, breast, colon | Pre-clinical | ^9^ |
|  | Conatumumab (AMG  655) | Ovarial | Pre-clinical | ^10^ |
|  | Vinorelbine and cisplatin | Advanced non-small cell lung cancer | III | ^11^ |
|  |  |  |  |  |
| CPT, circularly permuted TRAIL | Thalidomide | Multiple myeloma | II | ^12^ |
|  | None | Multiple myeloma | II | ^13^ |
|  | None | Relapsed or refractory multiple myeloma | Ib | ^14^ |
|  | Rocaglamide | Multiple myeloma and acute T-cell leukemia | Pre-clinical | ^15^ |
|  | | | | |
| TRAIL‑R agonistic antibodies |  |  |  |  |
| Mapatumumab  (Anti-TRAIL-R1 Ab) | None | Advanced solid tumours | Ia | ^16^ |
|  | None | Advanced solid tumours | Ia | ^17^ |
|  | Gemcitabine and cisplatin | Advanced solid tumours | I | ^18^ |
|  | None | Non-Hodgkin's lymphoma | Ib/II | ^19^ |
|  | Sorafenib | Hepatocellular | II | ^20^ |
|  | None | Non-small cell lung cancer | II | ^21^ |
|  | None | Colorectal | II | ^22^ |
|  | Paclitaxil and carboplatin | Non-small cell lung cancer | II R | ^23^ |
|  | Paclitaxil and carboplatin | Advanced solid tumours | I | ^24^ |
|  | Gemcitabine | Pancreatic | I | ^25^ |
|  | None | Advanced solid tumours | I | ^26^ |
|  |  |  |  |  |
| Conatumumab/AMG 655  (Anti-TRAIL-R2 Ab) | None | Advanced solid tumours | I | ^27^ |
|  | None | Non-small cell lung cancer, colorectal | Ia | ^26^ |
|  | Doxorubicin | Soft tissue sarcoma | II | ^28^ |
|  | Ganitumab (AMG 479) | Pancreatic | II | ^25^ |
|  | FOLFOX6 and bevacizumab | Colorectal | II | ^29^ |
|  | Ganitumab (AMG 479) and  FOLFIRI | Colorectal | II | ^30^ |
|  | Ganitumab (AMG 479) | Advanced, refractory solid tumours | I | ^31^ |
|  | Doxorubicin | Unresectable soft tissue sarcomas | I/II | ^28^ |
|  | Paclitaxil and carboplatin | Non-small cell lung cancer | II | ^32^ |
|  | Dulanermin | Lung, breast, colon | Pre-clinical | ^9^ |
|  | Dulanermin | Ovarial | Pre-clinical | ^10^ |
|  |  |  |  |  |
| Drozitumab (Anti-TRAIL-R2 Ab) | None | Advanced tumors | Ia | ^33^ |
|  | mFOLFOX6 and  bevacizumab | Colorectal | Ib | ^34^ |
|  |  |  |  |  |
| Lexatumumab  (Anti-TRAIL-R2 Ab) | None | Advanced solid tumors | Ia | ^35^ |
|  | None | Advanced solid tumors | Ib | ^36^ |
|  | None | Pediatric solid tumors | I | ^37^ |
|  |  |  |  |  |
| LBY135 (Anti-TRAIL-R2 Ab) | Capecitabine | Advanced solid tumors | I/II | ^38^ |
|  | Sorafenib | Hepatocellular | Pre-clinical | ^39^ |
|  | None | Chronic lymphocytic leukaemia | Pre-clinical | ^40^ |
|  |  |  |  |  |
| Tigatuzumab  (Anti-TRAIL-R2 Ab) | None | Refractory  Carcinoma, lymphoma | I | ^41^ |
|  | Gemcitabine | Pancreatic ductal adenocarcinoma | I | ^42^ |
|  | Sorafenib | Hepatocellular | II | ^43^ |
|  | Gemcitabine | Pancreatic | II | ^44^ |
|  | Paclitaxel | Breast | II | ^45^ |
|  | o-rMETase | Pancreatic | I | ^46^ |
|  | Paclitaxel | Breast | II | ^47^ |
|  | Carboplatin and paclitaxel | Non-small cell lung cancer | II | ^48^ |
|  | Dovitinib | Hepatocellular | Pre-clinical | ^49^ |

**References**

1 Ashkenazi, A. *et al.* Safety and antitumor activity of recombinant soluble Apo2 ligand. *The Journal of clinical investigation* **104**, 155-162, doi:10.1172/jci6926 (1999).

2 Daniel, D. *et al.* Cooperation of the proapoptotic receptor agonist rhApo2L/TRAIL with the CD20 antibody rituximab against non-Hodgkin lymphoma xenografts. *Blood* **110**, 4037-4046, doi:10.1182/blood-2007-02-076075 (2007).

3 Cheah, C. Y. *et al.* Dulanermin with rituximab in patients with relapsed indolent B-cell lymphoma: an open-label phase 1b/2 randomised study. *The Lancet. Haematology* **2**, e166-174, doi:10.1016/s2352-3026(15)00026-5 (2015).

4 Soria, J. C. *et al.* Phase 1b study of dulanermin (recombinant human Apo2L/TRAIL) in combination with paclitaxel, carboplatin, and bevacizumab in patients with advanced non-squamous non-small-cell lung cancer. *Journal of clinical oncology : official journal of the American Society of Clinical Oncology* **28**, 1527-1533, doi:10.1200/jco.2009.25.4847 (2010).

5 Soria, J. C. *et al.* Randomized phase II study of dulanermin in combination with paclitaxel, carboplatin, and bevacizumab in advanced non-small-cell lung cancer. *Journal of clinical oncology : official journal of the American Society of Clinical Oncology* **29**, 4442-4451, doi:10.1200/jco.2011.37.2623 (2011).

6 Wainberg, Z. A. *et al.* A phase 1B study of dulanermin in combination with modified FOLFOX6 plus bevacizumab in patients with metastatic colorectal cancer. *Clinical colorectal cancer* **12**, 248-254, doi:10.1016/j.clcc.2013.06.002 (2013).

7 Herbst, R. S. *et al.* Phase I dose-escalation study of recombinant human Apo2L/TRAIL, a dual proapoptotic receptor agonist, in patients with advanced cancer. *Journal of clinical oncology : official journal of the American Society of Clinical Oncology* **28**, 2839-2846, doi:10.1200/jco.2009.25.1991 (2010).

8 Pan, Y. *et al.* Evaluation of pharmacodynamic biomarkers in a Phase 1a trial of dulanermin (rhApo2L/TRAIL) in patients with advanced tumours. *British journal of cancer* **105**, 1830-1838, doi:10.1038/bjc.2011.456 (2011).

9 Graves, J. D. *et al.* Apo2L/TRAIL and the death receptor 5 agonist antibody AMG 655 cooperate to promote receptor clustering and antitumor activity. *Cancer cell* **26**, 177-189, doi:10.1016/j.ccr.2014.04.028 (2014).

10 Tuthill, M. H. *et al.* TRAIL-R2-specific antibodies and recombinant TRAIL can synergise to kill cancer cells. *Oncogene* **34**, 2138-2144, doi:10.1038/onc.2014.156 (2015).

11 Ouyang, X. *et al.* Phase III study of dulanermin (recombinant human tumor necrosis factor-related apoptosis-inducing ligand/Apo2 ligand) combined with vinorelbine and cisplatin in patients with advanced non-small-cell lung cancer. *Investigational new drugs* **36**, 315-322, doi:10.1007/s10637-017-0536-y (2018).

12 Geng, C. *et al.* A multicenter, open-label phase II study of recombinant CPT (Circularly Permuted TRAIL) plus thalidomide in patients with relapsed and refractory multiple myeloma. *American journal of hematology* **89**, 1037-1042, doi:10.1002/ajh.23822 (2014).

13 Leng, Y. *et al.* Phase II open-label study of recombinant circularly permuted TRAIL as a single-agent treatment for relapsed or refractory multiple myeloma. *Chinese journal of cancer* **35**, 86, doi:10.1186/s40880-016-0140-0 (2016).

14 Hou, J. *et al.* A Phase1b Dose Escalation Study of Recombinant Circularly Permuted TRAIL in Patients With Relapsed or Refractory Multiple Myeloma. *American journal of clinical oncology* **41**, 1008-1014, doi:10.1097/coc.0000000000000404 (2018).

15 Wu, Y. *et al.* Rocaglamide breaks TRAIL-resistance in human multiple myeloma and acute T-cell leukemia in vivo in a mouse xenogtraft model. *Cancer letters* **389**, 70-77, doi:10.1016/j.canlet.2016.12.010 (2017).

16 Tolcher, A. W. *et al.* Phase I pharmacokinetic and biologic correlative study of mapatumumab, a fully human monoclonal antibody with agonist activity to tumor necrosis factor-related apoptosis-inducing ligand receptor-1. *Journal of clinical oncology : official journal of the American Society of Clinical Oncology* **25**, 1390-1395, doi:10.1200/jco.2006.08.8898 (2007).

17 Hotte, S. J. *et al.* A phase 1 study of mapatumumab (fully human monoclonal antibody to TRAIL-R1) in patients with advanced solid malignancies. *Clinical cancer research : an official journal of the American Association for Cancer Research* **14**, 3450-3455, doi:10.1158/1078-0432.Ccr-07-1416 (2008).

18 Mom, C. H. *et al.* Mapatumumab, a fully human agonistic monoclonal antibody that targets TRAIL-R1, in combination with gemcitabine and cisplatin: a phase I study. *Clinical cancer research : an official journal of the American Association for Cancer Research* **15**, 5584-5590, doi:10.1158/1078-0432.Ccr-09-0996 (2009).

19 Younes, A. *et al.* A Phase 1b/2 trial of mapatumumab in patients with relapsed/refractory non-Hodgkin's lymphoma. *British journal of cancer* **103**, 1783-1787, doi:10.1038/sj.bjc.6605987 (2010).

20 Ciuleanu, T. *et al.* A randomized, double-blind, placebo-controlled phase II study to assess the efficacy and safety of mapatumumab with sorafenib in patients with advanced hepatocellular carcinoma. *Annals of oncology : official journal of the European Society for Medical Oncology* **27**, 680-687, doi:10.1093/annonc/mdw004 (2016).

21 Greco, F. A. *et al.* Phase 2 study of mapatumumab, a fully human agonistic monoclonal antibody which targets and activates the TRAIL receptor-1, in patients with advanced non-small cell lung cancer. *Lung cancer (Amsterdam, Netherlands)* **61**, 82-90, doi:10.1016/j.lungcan.2007.12.011 (2008).

22 Trarbach, T. *et al.* Phase II trial of mapatumumab, a fully human agonistic monoclonal antibody that targets and activates the tumour necrosis factor apoptosis-inducing ligand receptor-1 (TRAIL-R1), in patients with refractory colorectal cancer. *British journal of cancer* **102**, 506-512, doi:10.1038/sj.bjc.6605507 (2010).

23 von Pawel, J. *et al.* Phase II trial of mapatumumab, a fully human agonist monoclonal antibody to tumor necrosis factor-related apoptosis-inducing ligand receptor 1 (TRAIL-R1), in combination with paclitaxel and carboplatin in patients with advanced non-small-cell lung cancer. *Clinical lung cancer* **15**, 188-196.e182, doi:10.1016/j.cllc.2013.12.005 (2014).

24 Leong, S. *et al.* Mapatumumab, an antibody targeting TRAIL-R1, in combination with paclitaxel and carboplatin in patients with advanced solid malignancies: results of a phase I and pharmacokinetic study. *Journal of clinical oncology : official journal of the American Society of Clinical Oncology* **27**, 4413-4421, doi:10.1200/jco.2008.21.7422 (2009).

25 Kindler, H. L. *et al.* A randomized, placebo-controlled phase 2 study of ganitumab (AMG 479) or conatumumab (AMG 655) in combination with gemcitabine in patients with metastatic pancreatic cancer. *Annals of oncology : official journal of the European Society for Medical Oncology* **23**, 2834-2842, doi:10.1093/annonc/mds142 (2012).

26 Herbst, R. S. *et al.* A first-in-human study of conatumumab in adult patients with advanced solid tumors. *Clinical cancer research : an official journal of the American Association for Cancer Research* **16**, 5883-5891, doi:10.1158/1078-0432.Ccr-10-0631 (2010).

27 Doi, T. *et al.* Phase 1 study of conatumumab, a pro-apoptotic death receptor 5 agonist antibody, in Japanese patients with advanced solid tumors. *Cancer chemotherapy and pharmacology* **68**, 733-741, doi:10.1007/s00280-010-1544-1 (2011).

28 Demetri, G. D. *et al.* First-line treatment of metastatic or locally advanced unresectable soft tissue sarcomas with conatumumab in combination with doxorubicin or doxorubicin alone: a phase I/II open-label and double-blind study. *European journal of cancer (Oxford, England : 1990)* **48**, 547-563, doi:10.1016/j.ejca.2011.12.008 (2012).

29 Fuchs, C. S. *et al.* TRAIL receptor agonist conatumumab with modified FOLFOX6 plus bevacizumab for first-line treatment of metastatic colorectal cancer: A randomized phase 1b/2 trial. *Cancer* **119**, 4290-4298, doi:10.1002/cncr.28353 (2013).

30 Cohn, A. L. *et al.* A randomized, placebo-controlled phase 2 study of ganitumab or conatumumab in combination with FOLFIRI for second-line treatment of mutant KRAS metastatic colorectal cancer. *Annals of oncology : official journal of the European Society for Medical Oncology* **24**, 1777-1785, doi:10.1093/annonc/mdt057 (2013).

31 Tabernero, J. *et al.* Ramucirumab versus placebo in combination with second-line FOLFIRI in patients with metastatic colorectal carcinoma that progressed during or after first-line therapy with bevacizumab, oxaliplatin, and a fluoropyrimidine (RAISE): a randomised, double-blind, multicentre, phase 3 study. *The Lancet. Oncology* **16**, 499-508, doi:10.1016/s1470-2045(15)70127-0 (2015).

32 Paz-Ares, L. *et al.* A randomized phase 2 study of paclitaxel and carboplatin with or without conatumumab for first-line treatment of advanced non-small-cell lung cancer. *Journal of thoracic oncology : official publication of the International Association for the Study of Lung Cancer* **8**, 329-337, doi:10.1097/JTO.0b013e31827ce554 (2013).

33 Camidge, D. R. *et al.* A phase I safety and pharmacokinetic study of the death receptor 5 agonistic antibody PRO95780 in patients with advanced malignancies. *Clinical cancer research : an official journal of the American Association for Cancer Research* **16**, 1256-1263, doi:10.1158/1078-0432.Ccr-09-1267 (2010).

34 Rocha Lima, C. M. *et al.* Phase Ib study of drozitumab combined with first-line mFOLFOX6 plus bevacizumab in patients with metastatic colorectal cancer. *Cancer investigation* **30**, 727-731, doi:10.3109/07357907.2012.732163 (2012).

35 Plummer, R. *et al.* Phase 1 and pharmacokinetic study of lexatumumab in patients with advanced cancers. *Clinical cancer research : an official journal of the American Association for Cancer Research* **13**, 6187-6194, doi:10.1158/1078-0432.Ccr-07-0950 (2007).

36 Wakelee, H. A. *et al.* Phase I and pharmacokinetic study of lexatumumab (HGS-ETR2) given every 2 weeks in patients with advanced solid tumors. *Annals of oncology : official journal of the European Society for Medical Oncology* **21**, 376-381, doi:10.1093/annonc/mdp292 (2010).

37 Merchant, M. S. *et al.* Phase I trial and pharmacokinetic study of lexatumumab in pediatric patients with solid tumors. *Journal of clinical oncology : official journal of the American Society of Clinical Oncology* **30**, 4141-4147, doi:10.1200/jco.2012.44.1055 (2012).

38 Sharma, S. *et al.* Safety, pharmacokinetics, and pharmacodynamics of the DR5 antibody LBY135 alone and in combination with capecitabine in patients with advanced solid tumors. *Investigational new drugs* **32**, 135-144, doi:10.1007/s10637-013-9952-9 (2014).

39 Chen, K. F. *et al.* Sorafenib overcomes TRAIL resistance of hepatocellular carcinoma cells through the inhibition of STAT3. *Clinical cancer research : an official journal of the American Association for Cancer Research* **16**, 5189-5199, doi:10.1158/1078-0432.Ccr-09-3389 (2010).

40 Natoni, A. *et al.* TRAIL signals to apoptosis in chronic lymphocytic leukaemia cells primarily through TRAIL-R1 whereas cross-linked agonistic TRAIL-R2 antibodies facilitate signalling via TRAIL-R2. *British journal of haematology* **139**, 568-577, doi:10.1111/j.1365-2141.2007.06852.x (2007).

41 Forero-Torres, A. *et al.* Phase I trial of weekly tigatuzumab, an agonistic humanized monoclonal antibody targeting death receptor 5 (DR5). *Cancer biotherapy & radiopharmaceuticals* **25**, 13-19, doi:10.1089/cbr.2009.0673 (2010).

42 Rajeshkumar, N. V. *et al.* A combination of DR5 agonistic monoclonal antibody with gemcitabine targets pancreatic cancer stem cells and results in long-term disease control in human pancreatic cancer model. *Molecular cancer therapeutics* **9**, 2582-2592, doi:10.1158/1535-7163.Mct-10-0370 (2010).

43 Cheng, A. L. *et al.* Safety and efficacy of tigatuzumab plus sorafenib as first-line therapy in subjects with advanced hepatocellular carcinoma: A phase 2 randomized study. *Journal of hepatology* **63**, 896-904, doi:10.1016/j.jhep.2015.06.001 (2015).

44 Forero-Torres, A. *et al.* Phase 2, multicenter, open-label study of tigatuzumab (CS-1008), a humanized monoclonal antibody targeting death receptor 5, in combination with gemcitabine in chemotherapy-naive patients with unresectable or metastatic pancreatic cancer. *Cancer medicine* **2**, 925-932, doi:10.1002/cam4.137 (2013).

45 Forero-Torres, A. *et al.* TBCRC 019: A Phase II Trial of Nanoparticle Albumin-Bound Paclitaxel with or without the Anti-Death Receptor 5 Monoclonal Antibody Tigatuzumab in Patients with Triple-Negative Breast Cancer. *Clinical cancer research : an official journal of the American Association for Cancer Research* **21**, 2722-2729, doi:10.1158/1078-0432.Ccr-14-2780 (2015).

46 Yamamoto, J. *et al.* Oral recombinant methioninase increases TRAIL receptor-2 expression to regress pancreatic cancer in combination with agonist tigatuzumab in an orthotopic mouse model. *Cancer letters*, doi:10.1016/j.canlet.2020.07.034 (2020).

47 Paoletti, C. *et al.* Significance of Circulating Tumor Cells in Metastatic Triple-Negative Breast Cancer Patients within a Randomized, Phase II Trial: TBCRC 019. *Clinical cancer research : an official journal of the American Association for Cancer Research* **21**, 2771-2779, doi:10.1158/1078-0432.Ccr-14-2781 (2015).

48 Reck, M. *et al.* A randomized, double-blind, placebo-controlled phase 2 study of tigatuzumab (CS-1008) in combination with carboplatin/paclitaxel in patients with chemotherapy-naïve metastatic/unresectable non-small cell lung cancer. *Lung cancer (Amsterdam, Netherlands)* **82**, 441-448, doi:10.1016/j.lungcan.2013.09.014 (2013).

49 Chen, K. F. *et al.* Dovitinib sensitizes hepatocellular carcinoma cells to TRAIL and tigatuzumab, a novel anti-DR5 antibody, through SHP-1-dependent inhibition of STAT3. *Biochemical pharmacology* **83**, 769-777, doi:10.1016/j.bcp.2011.12.035 (2012).
